# When Blood Meets Nitrogen Oxides: Pregnancy Complications and Air Pollution Exposure

**DOI:** 10.1289/ehp.121-a136

**Published:** 2013-04-01

**Authors:** Tanya Tillett

**Affiliations:** Tanya Tillett, MA, of Durham, NC, is a staff writer/editor for *EHP*. She has been on the *EHP* staff since 2000 and has represented the journal at national and international conferences.

A number of studies have documented associations between maternal air pollution exposure and adverse birth outcomes such as low birth weight. However, only limited information exists for air pollution vis-à-vis pregnancy complications, which may share some underlying mechanisms with poor birth outcomes. A new study now reports an association between exposure to nitrogen oxides (NO_X_) and two pregnancy complications: gestational diabetes and preeclampsia (high blood pressure and protein in the urine) [*EHP* 121(4):488–493; http://dx.doi.org/1205736].

The study included women who gave birth during 1999–2005 in Scania, a county in southern Sweden. The women were included in the Swedish Medical Birth Registry, which consists of almost all infants born in Sweden; they comprised 81,110 singleton births, including 1,599 cases of gestational diabetes and 2,370 cases of preeclampsia.

The investigators estimated mean monthly and trimester NO_X_ exposures for each birth by combining emissions data with meteorological data. In addition, they used road traffic data from the Swedish National Road Database to calculate each woman’s proximity during pregnancy to heavily traveled roads. Gestational diabetes was assessed in relation to first or second trimester exposure to NO_X_, and preeclampsia was assessed in relation to NO_X_ exposure during all three trimesters.

**Figure:**
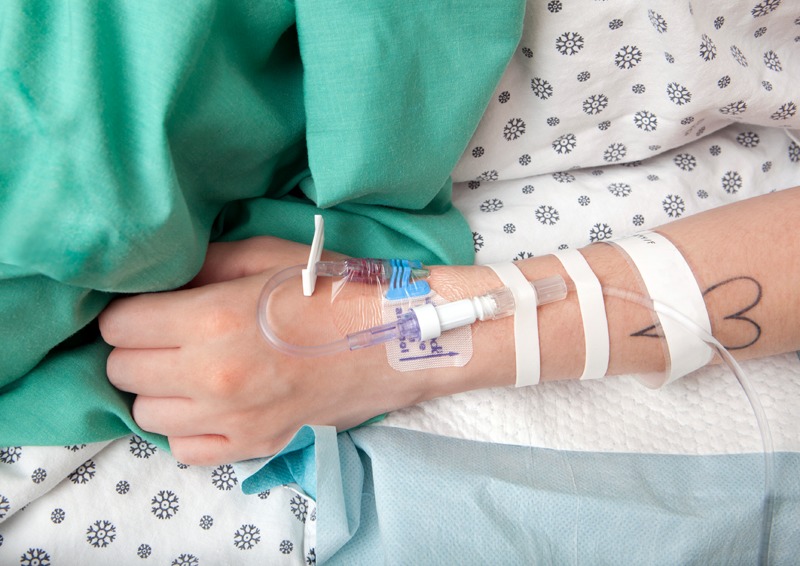
Gestational diabetes is treated through diet and exercise. The only cure for preeclampsia is delivery; in severe cases, obstetricians will induce labor or perform a cesarean section. Both gestational diabetes and preeclampsia are potentially serious complications. © Sarah Small/Getty Images

Air pollution is relatively low in Scania, with ambient levels of NO_2_ (the dominant constituent of NO_X_ and a proxy for the NO_X_ family of compounds) well below the 40-µg/m^3^ annual standard set by the World Health Organization. In this study, the level of modeled NO_X_ exposure averaged 16.4 µg/m^3^ over the entire study period, decreasing from 18.2 µg/m^3^ in 1999 to 13.3 µg/m^3^ in 2005.

The researchers found an increased prevalence of gestational diabetes with each quartile of NO_X_ exposure during the second trimester. Gestational diabetes was more common in urban versus nonurban subjects, but was increased among both urban and nonurban women with NO_X_ above the average concentration for each population. Preeclampsia also was associated with increasingly higher levels of NO_X_ exposure. The data suggested an association between gestational diabetes and proximity to high-traffic roads, although no obvious association was observed between traffic density and preeclampsia.

The large sample size and individual NO_X_ exposure estimates were strengths of the study, but confounding by factors related to socioeconomic status and other unmeasured characteristics could not be ruled out. The authors note that associations with the highest level of exposure were comparable in magnitude to those of other well-known risk factors such as obesity or being over 35 years of age. These findings, if reinforced with further research, could suggest the need to reduce exposures to pregnant women below current limits.

